# Proof-of-Concept of the Virtual Reality Comprehensive Balance Assessment and Training for Sensory Organization of Dynamic Postural Control

**DOI:** 10.3389/fbioe.2021.678006

**Published:** 2021-07-29

**Authors:** Sanghee Moon, Chun-Kai Huang, Maryam Sadeghi, Abiodun E. Akinwuntan, Hannes Devos

**Affiliations:** ^1^Department of Physical Therapy, Ithaca College, Ithaca, NY, United States; ^2^Laboratory for Advanced Rehabilitation Research in Simulation, Department of Physical Therapy, Rehabilitation Science, and Athletic Training, University of Kansas Medical Center, Kansas City, KS, United States; ^3^Office of the Dean, School of Health Professions, University of Kansas Medical Center, Kansas City, KS, United States

**Keywords:** virtual reality, balance, sensory organization test, postural control, center of pressure

## Abstract

Accurate quantification of the impact of visual, somatosensory, and vestibular systems on postural control may inform tailor-made balance intervention strategies. The aim of this proof-of-concept study was to determine the safety, sense of presence, system usability, and face validity of a newly developed Virtual Reality Comprehensive Balance Assessment and Training (VR-ComBAT) in healthy young individuals. The VR-ComBAT included six balance condition: (1) stable surface with fixed virtual reality (VR) surroundings; (2) stable surface with blacked out VR surroundings; (3) stable surface with VR visual conflict; (4) unstable surface with fixed VR surroundings; (5) unstable surface with blacked out VR surroundings; and (6) unstable surface with VR visual conflict. Safety was evaluated using the number of adverse events, including scores on the Simulator Sickness Questionnaire. Sense of presence was evaluated using the igroup Presence Questionnaire (iPQ). System usability was assessed using the Systems Usability Scale (SUS). Friedman analyses with *post hoc* Wilcoxon Signed Rank tests were employed to demonstrate face validity by quantifying center of pressure (COP) changes in mean distance, mean velocity, and mean frequency in the anteroposterior (AP) and mediolateral (ML) direction across the six conditions. Twenty-three participants (27.4 ± 8.0 years old; 13 women) reported no adverse events. Participants scores on average 44.9 ± 9.6 on the iPQ and 79.7 ± 9.9 on the SUS. *Post hoc* analyses showed significant changes in COP-based measures when compared to baseline. The mean frequency change of COP showed direction-dependence in which increased frequency change in AP was observed while decreased change in ML was noted. The VR-ComBAT provides a safe, feasible, and cost-effective VR environment that demonstrates consistent sensory re-weighting between visual, somatosensory, and vestibular systems. Future studies should investigate whether VR-ComBAT can be used to inform precision rehabilitation of balance and fall prevention in older adults without and with neurological conditions.

## Introduction

Postural control requires the brain to integrate information from vision, somatosensory, and vestibular cues in order to respond timely and accurately to changes in the body’s alignment and tone with respect to visual surroundings, support surface, internal references, and gravity (Alcock et al., 2018; Ivanenko and Gurfinkel, 2018). These sensory systems sometimes provide conflicting information (Nashner and Peters, 1990). To illustrate, while sitting on a moving bus, the eyes inform the brain that the surroundings appear to be moving whereas the somatosensory system provides information that the body is stationary. In such cases, the brain needs to ignore or prioritize conflicting information to accomplish postural control (Gaerlan, 2010). Discrepancies in these systems become even more apparent in individuals with neurological conditions, resulting in increased postural imbalance, a higher risk of falls, and, in many cases, a higher fear of falling (Horak, 2006; Cronin et al., 2017). Accurate detection of the system that affects postural control in neurological conditions is key to tailor balance intervention and fall prevention programs to the needs of individuals at risk of falls.

Several computerized balance assessment tools have been developed, including dynamic posturography [e.g., Neurocom^®^ SMART EquiTest^®^, Bertec^®^ computerized dynamic posturography (CDP/IVR), Biodex^®^ Balance] or wearable motion sensors (e.g., APDM Opal). These computerized balance assessment tools enable quantitative assessment of postural control (Mancini and Horak, 2010), reduce test performance variability, increase sensitivity to subtle changes (Visser et al., 2008), and determine the systems that may underly impaired postural control (Mancini and Horak, 2010). Of those, the Neurocom^®^ SMART EquiTest^®^ system (Natus, San Carlos, CA, United States) and Bertec computerized dynamic posturography (Bertec^®^, Columbus, OH, United States) are considered the gold standard of dynamic posturography assessment. These computerized sensory organization tests (SOT) challenge the visual, somatosensory, and vestibular systems to provide a detailed assessment of the underlying sensory deficits affecting balance. Although effective in evaluating and treating balance disorders (Alahmari et al., 2014), the high cost ($80,000–$180,000), space needs for the equipment, lack of portability, and time needed for training, have limited the use of the computerized SOT in clinical practice (Visser et al., 2008). The Clinical Test of Sensory Interaction on Balance (CTSIB) was developed to discern the relative contributions of the visual, somatosensory, and vestibular systems to postural control (Shumway-Cook and Horak, 1986). This test includes six static balance conditions with eyes open, eyes closed, and the use of a dome to create visual conflict combining with feet on firm or foam surface. This test was later modified to only four conditions, excluding the visual dome from the test procedures as the balance tasks with the visual dome did not differ from those in the eyes closed conditions (Cohen et al., 1993). Although clinically useful, the scoring system is crude, semi-objective, and may not be sensitive enough to detect subtle changes in postural control in neurodegenerative conditions (Suttanon et al., 2011).

The advent of portable force platforms with head-mounted virtual reality (VR) technology may provide a clinical, cost-effective, and user-friendly dynamic posturography assessment without compromising the evaluative and rehabilitative effectiveness of current computerized SOT. Although VR technology has emerged in the rehabilitation realm as a promising intervention tool to improve balance and gait (de Rooij et al., 2016; Cano Porras et al., 2018, 2019; Massetti et al., 2018; Chen et al., 2020), only few studies utilized VR to assess the sensory organization of postural balance (Lubetzky et al., 2018, 2019; Trueblood et al., 2018; Wittstein et al., 2020). However, it is not clear whether the SOT administered using a head-mounted VR set is safe and easy to use, provides a realistic experience to the user, and discriminates between postural control conditions that test different balance systems.

Unlike the Equilibrium Score calculated from the force platform of the Equitest^®^ that evaluates postural balance through center of pressure (COP) displacement only in the anteroposterior (AP) direction, portable force platforms provide a multidimensional assessment of postural control. Quiet standing on a foam increased COP displacement in the AP direction by 8%, and in the mediolateral (ML) direction by 21% (Reynard et al., 2019). Adding assessment of ML sway and sway area has shown to quantify fall risk in neurological conditions, such as changes in postural sway characteristics while standing with eyes closed in Parkinson’s disease (Błaszczyk et al., 2007). Additionally, measuring COP frequency in AP and ML directions brings perspective of adopting different strategies to maintain balance. For example, evidence suggests that changes in perturbation conditions alter COP AP or ML frequency characteristics (Creath et al., 2005). Previous study also noted that postural sway in AP and ML directions can be attributed to ankle and hip control, respectively (Saffer et al., 2008).

The aim of this proof-of-concept study was to demonstrate the safety, sense of presence, system usability, and face validity of the Virtual Reality Comprehensive Balance Assessment and Training (VR-ComBAT) in healthy individuals. The overall hypothesis was that the VR-ComBAT will provide a safe and standardized VR-based balance testing environment in the healthy population. To test our hypothesis, we gauged sense of presence and system usability in the VR environment and satisfaction with our novel VR-based test. Face validity of the VR-ComBAT was evaluated by comparing multidirectional COP displacement and frequency measures across different postural control conditions.

## Materials and Equipment

### Hardware and Software Specifications

The VR-ComBAT consists of a computer that processes the VR input and output [Alienware (Intel^®^ Core^TM^ i7-7800X CPU @ 3.50GHz; 16.0 GB RAM), Dell USA Corporation, Round Rock, TX, United States], a commercially available, head-mounted device (HMD) with integrated VR (HTC VIVE Pro Eye, HTC, Taoyuan, Taiwan), and two VR tracking sensors (Steam VR Base Stations, HTC, Taoyuan, Taiwan). The HTC VIVE Pro Eye headset includes dual-OLED 3.5-inch displays with a combined resolution of 2,880 × 1,600 pixels. The refresh rate of the screen is 90 Hz. The field of view is 110 degrees. The Steam VR (version 1.13, Valve, Bellevue, WA, United States) was used to simultaneously link the computer and the VR headset.

**FIGURE 1:**
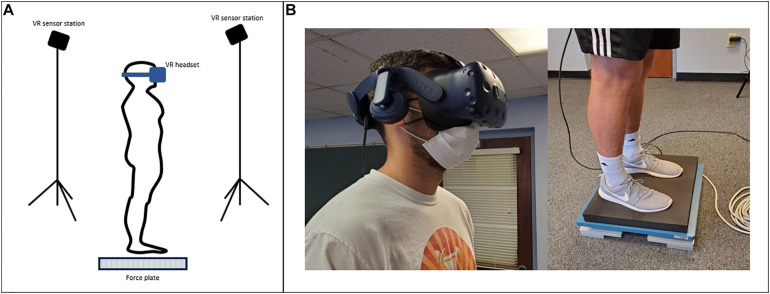
**(A)** Virtual Reality Comprehensive Balance Assessment and Training (VR-ComBAT) setup; **(B)** A participant wears the VR headset and stands on the foam surface placed over the force plate.

The VR headset was integrated with a force plate (AMTI Optima, Watertown, MA, United States) to measure the displacement and velocity of COP. The sampling frequency of the force plate was 200 Hz. The force plate was manually synchronized with the VR system by simultaneously starting the VR conditions and force plate measurement at each trial. The experimental setup is shown in Figure 1.

**FIGURE 2:**
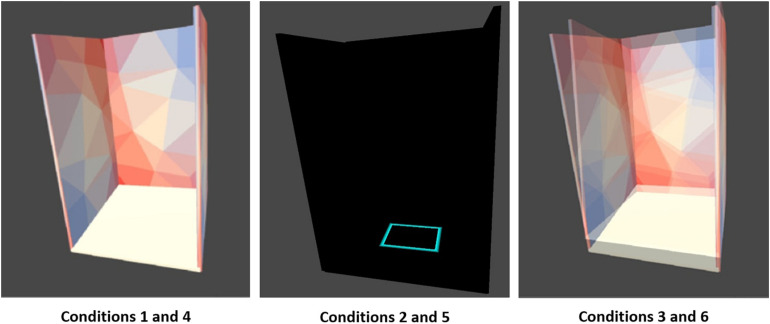
Six VR-ComBAT conditions. (1) stable surface with fixed virtual reality (VR) surroundings; (2) stable surface with blacked out VR surroundings; (3) stable surface with VR visual conflict; (4) unstable surface with fixed VR surroundings; (5) unstable surface with blacked out VR surroundings; and (6) unstable surface with VR visual conflict.

Unity 3D (version 2019.3.0; San Francisco, CA, United States) was utilized to create the VR-ComBAT environment. The executable application is available at http://bit.ly/VR-ComBAT (github). The basic setup of the VR environment includes three panels (one front, and two side panels), positioned at 90-degree angle to each, with multi-colored triangular patterns to help participants with visual fixation on the VR environment (Figure 2).

### Balance Conditions

The VR-ComBAT emulates six different conditions, that, in combination, test visual, somatosensory, and vestibular systems of balance (Figure 2). These conditions mimic the six conditions in the SOT of the Equitest^®^: (1) stable surface with fixed VR surrounding; (2) stable surface with blacked out VR surroundings; (3) stable surface with VR visual conflict; (4) unstable surface with fixed VR surroundings; (5) unstable surface with blacked out VR surroundings; and (6) unstable surface with VR visual conflict.

Condition 1 is a baseline that measures static balance on a fixed surface. The surrounding panels in the VR environment remain stationary. Participants can use input from visual, somatosensory, and vestibular systems to maintain balance. In condition 2, participants cannot rely on vision to remain upright since the VR surroundings are blackened out. Condition 3 creates a conflict between normal input from the somatosensory and vestibular systems and the visual information from the moving VR panels. The surrounding panels are moving in the anteroposterior direction with a maximum of 20 degrees and a maximum velocity of 15 degrees/s. Conditions 4, 5, and 6 are identical to 1, 2, and 3, respectively. However, in conditions 4, 5, and 6, a foam (Amazon Basics Balance Pad for Exercise Training, 35 cm × 5 cm × 40 cm, density = 0.04 g/cm^3^) is placed between the feet and the force plate, thus challenging the somatosensory system.

## Methods

### Participants

This study was conducted in the Laboratory for Advanced Rehabilitation Research in Simulation, at the University of Kansas Medical Center. This proof-of-concept study recruited 23 healthy participants if they were (1) between 19 and 65 years of age; (2) had no walking or balance impairment [>45 in Berg Balance Scale (BBS; Berg et al., 1992) and >20 in Mini Balance Evaluation Systems Test (Mini-BESTest; Leddy et al., 2011)]; and (3) were able to understand and follow instructions in English. We excluded individuals who (1) had a history of neurological or vestibular conditions and (2) had visual acuity or visual field impairment that could not be resolved by corrective lenses. All participants signed written informed consent. The study ethics was approved by the Institutional Review Board at the University of Kansas Medical Center (#STUDY00145395).

### Study Protocol

**FIGURE 3:**
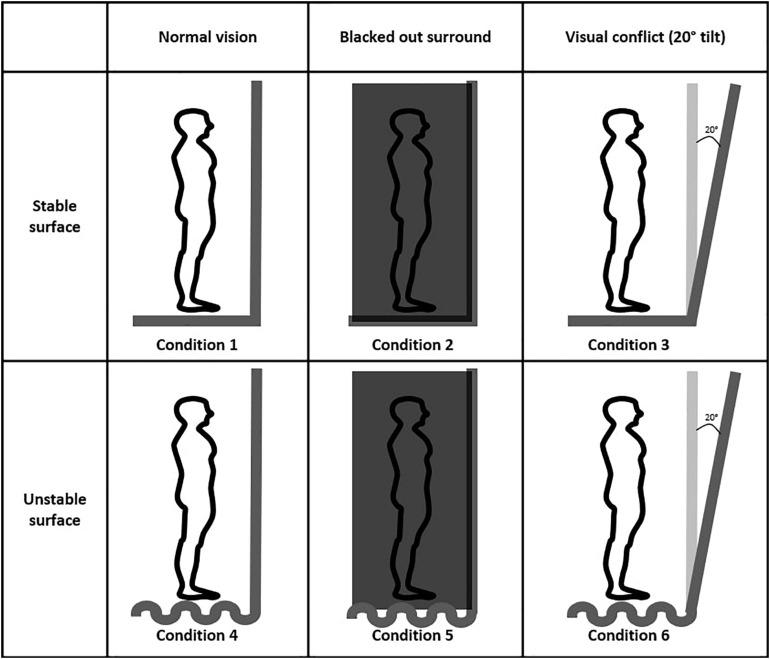
Six VR-ComBAT conditions in the virtual environment. Conditions 1 and 4, conditions 2 and 5, and conditions 3 and 6 share the same VR balance testing environments.

**FIGURE 4:**
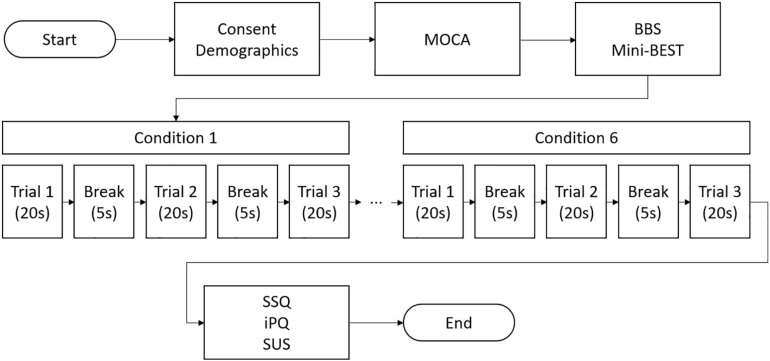
A flow chart of VR-ComBAT experimental procedure (BBS = Berg Balance Scale; iPQ = igroup Presence Questionnaire; Mini-BEST = Mini Balance Evaluation Systems Test; MOCA = Montreal Cognitive Assessment; SSQ = Simulator Sickness Questionnaire; SUS = System Usability Scale).

Following written consent, demographic information (age, sex, and education) was collected. The BBS (total score: 56, lower score indicating higher risk of balance problems) and Mini-BESTest (total score: 28, lower score indicating the higher risk of balance problems) were administered to confirm the absence of any balance impairments. Cognitive impairments were ruled out using the Montreal Cognitive Assessment (MOCA; Nasreddine et al., 2005). Next, participants were fitted with the VR headset and asked to step on the force plate. Then, participants stood on the force plate (conditions 1–3) or on the foam surface (conditions 4–6) with their feet shoulder width apart and hands naturally placed at their sides. Participants were asked to remain in the same position during each VR-ComBAT task (Figure 3). All participants were newly recruited and had no previous exposure to the VR-ComBAT system. There was no practice or learning trial before the recording, since all the conditions in VR-ComBAT were thoroughly explained by the lead researcher and were easy to perform for healthy young participants. Participants with glasses wore the VR headset without taking their glasses off, which allowed participants to experience the VR environment with their normal vision or corrected-to-normal vision. Participants were instructed to stand as still as possible. Each condition consisted of three trials that lasted 20 s per trial. Between each trial, a 5-s break was given. For conditions 2 and 4 (blacked out VR surroundings), the light of the testing room was also turned off to create a completely dark VR environment by removing any lights coming from the gaps of the VR headset. During the experiment, safety was assured by a gait belt and a research team member next to a participant in case of a fall or near-fall event. The experimental protocol is shown in Figure 4.

### Outcome Measures

#### Safety

The number of adverse events (e.g., falls) was recorded. VR HMD is believed to increase simulator sickness (similar to motion sickness) compared to remote VR displays (Kim et al., 2014; Dennison et al., 2016). This increased proneness to simulator sickness is thought to stem from the high-fidelity, stereoscopic rendering of VR images. The realistic images displayed in VR HMD can create discrepancies between the perceived and expected visual sensory information, leading to increased symptoms of simulator sickness (Chang et al., 2020). We administered the Simulator Sickness Questionnaire (SSQ) after condition 6, which is the condition that was expected to induce most simulator sickness. The SSQ is regarded as the current gold standard in calculating simulator sickness in research (Kennedy et al., 1993; Jinjakam and Hamamoto, 2012). The SSQ is accurate and reliable to measure simulator sickness in high-fidelity VR environments such as driving simulators, flight simulators, and other VR systems (Kennedy et al., 1993). The score is comprised of three subsections, each with seven symptoms, in which there is some overlap: disorientation, nausea, and oculomotor. The symptoms include general discomfort, fatigue, headache, eyestrain, difficulty focusing, increased salivation, sweating, nausea, difficulty concentrating, the fullness of head, blurred vision, dizziness (eyes open and closed), vertigo, stomach awareness, and burping. The weighted score formula was used to calculate the global index, which reflects the total discomfort level, as well as the scores for the three subsections.

According to previous research (Kennedy et al., 1993), the three subscale scores were calculated by summing scores associated with each subscale and multiplying them by an appropriate weighting factor (9.54 for SSQ-Nausea, 7.58 for SSQ-Oculomotor, and 13.92 for SSQ-Disorientation). To calculate the total score of SSQ, the result was equal to the sum of the three unweighted subscale scores, multiplied by 3.74. A person with a total score of >100 is considered actively ill due to simulator sickness (Kennedy et al., 1993).

#### Sense of Presence

Sense of presence was evaluated using the iPQ. The iPQ is a multidimensional scale assessing the sense of presence in a VR environment. Sense of presence refers to the subjective feeling of being in a virtual environment. The reliability and validity of the iPQ have been established in previous work (Panahi et al., 2009). The 14 items of the questionnaire are scores on questionnaire contains 14 items, scored on an ordinal scale ranging from 1 to 5. One item reflects general sense of presence. The other items are categorized into sense of spatial presence (5 items), involvement (4 items), and experienced realism (4 items) (Hein et al., 2018).

#### System Usability

The SUS was administered to capture participants’ viewpoints on effectiveness, efficiency, and satisfaction levels of the VR-ComBAT (Brooke, 1996; Bangor et al., 2008). The questionnaire contains 10 items, each scored on a range from ranges from 0 to 100. The SUS total scores representing a composed measure of the system’s overall usability on a scale from 0 to 100.

#### Face Validity

Face validity evaluates the extent to which the measured variable appears to adequately measure the conceptual variable (Thomas et al., 1992). The face validity of VR-ComBAT to discriminate between different postural control conditions was evaluated using quantitative COP measures. These COP data were extracted from the force plate and processed using the MATLAB application (MathWorks, Natick, MA, United States) to calculate the following outcomes (Prieto et al., 1996):

• Mean distance in the AP (MeanAP) or ML (MeanML) direction: The average distance in the AP/ML direction from the mean COP.
• Mean velocity in the AP (VelAP) or ML (VelML) direction: The average velocity of COP in the AP/ML direction.
• 95% confidence ellipse area (95%Area): The area that encloses approximately 95% of the points on the COP path.
• Mean frequency in the AP (MfAP) or ML (MfML) direction: The frequency (Hz) of a sinusoidal oscillation with an average value of total path length of excursions in AP (or ML) over MeanAP (or MeanML).

### Statistical Analysis

All statistical analyses were performed using SPSS (version 26, IBM Corporation, Armonk, NY, United States). The distribution of data was examined for normality using the Shapiro–Wilk test. Since the number of participants of this study was small and almost all data were not normally distributed, the Friedman test was conducted to examine the differences between conditions followed by *post hoc* Wilcoxon Signed Rank test. We identified *a priori* four pairwise comparisons to minimize committing type 1 error. We compared (1) condition 2 to condition 1 to tease out somatosensory system contributions; (2) condition 4 to condition 1 to tease out visual system contributions; and (3) condition 5 to condition 1 and (4) condition 6 to condition 5 to tease out vestibular system contributions (Shumway-Cook, 2011; Pletcher et al., 2017). The effect size ($r=\frac{Z}{\sqrt{n}}$) was adopted to indicate the strength of two conditions of VR-ComBAT. Of note, given that Wilcoxon Signed-rank test was conducted in this study, to more accurately reflect this non-parametric approach, we calculated the effect size (*r*) according to Rosenthal et al. instead of Cohen’s *d* effect size (Rosenthal et al., 1994). The significance levels of all analyses were set α = 0.05.

## Results

Participants (13 women and 10 men) were on average 27.4 ± 8 years old and reported 19.2 ± 2.5 years of education. None of the participants showed any impairment in static or dynamic balance, as evidenced by maximum scores on the BBS (56 ± 0) and on the Mini-BESTest (28 ± 0). Participants scored on average 28.3 ± 1.4 on the MOCA, indicating no cognitive impairments (Table 1).

**TABLE 1:** Demographic and clinical characteristics.

	**Age**	**Sex**	**BBS**	**Mini-BESTest**	**MOCA**
Participants (*N* = 23)	27.4 ± 8.0	13 female, 10 male	56 ± 0	28 ± 0	28.3 ± 1.4

*BBS, Berg Balance Scale; Mini-BESTest, Mini Balance Evaluation Systems Test; MOCA, Montreal Cognitive Assessment.*

### Safety

No adverse events were reported during or after the study visit. SSQ total scores were on average 33.3 ± 66.1. Average SSQ average subscores were 2.9 ± 6.1 for nausea, 3 ± 5.9 for oculomotor, and 3 ± 7.2 for disorientation. Participants either scored none or slight on each item of the SSQ.

### Sense of Presence

The average iPQ score of total iPQ was 44.9 ± 9.6 with average subscores of 18.8 ± 3.7 for spatial presence, 11.3 ± 3.5 for involvement, and 10.7 ± 2.7 for experienced realism.

### System Usability

The SUS demonstrated nearly a total score of 80 in system usability (79.7 ± 9.9).

### Face Validity

The Friedman test revealed significant differences across the six conditions for all COP-based measures (*p* < 0.01). *Post hoc* analyses (Table 2) showed that participants exhibited worse performance on most COP measures in conditions 2, 4, and 5 compared to condition 1.

**TABLE 2:** COP-based measures across the six VR-ComBAT conditions (*N* = 23).

	**Condition 1**	**Condition 2**	**Condition 3**	Condition 4*	Condition 5*	**Condition 6**
MeanAP (mm)	3.71 (1.02)	4.17 (1.90) *0.21 [0.12–0.36]*	3.90 (1.26) *0.18 [0.13–0.22]*	5.50 (1.57) *0.61 [0.49–0.74]*	5.92 (1.83) *0.62 [0.78–0.46]*	5.92 (2.16) *0.62 [0.46–0.78]*
MeanML (mm)	1.33 (0.65)	1.61 (0.82)* *0.34 [0.3–0.38]*	1.73 (1.41) *0.37 [0.32–0.42]*	3.15 (1.08) *0.62 [0.49–0.75]*	3.24 (1.19) *0.62 [0.48–0.75]*	3.12 (1.19) *0.62 [0.49–0.75]*
VelAP (mm/s)	17.31 (3.46)	18.71 (4.03)* *0.4 [0.25–0.56]*	18.89 (3.22) *0.42 [0.25–0.58]*	29.73 (7.56) *0.62 [–0.25 to 1.49]*	32.96 (8.19) *0.62 [–0.49 to 1.72]*	29.59 (7.61)** *0.62 [–0.24 to 1.49]*
VelML (mm/s)	9.22 (1.21)	9.82 (1.65)* *0.38 [0.31–0.45]*	9.56 (1.74) *0.20 [0.12–0.27]*	15.40 (3.93) *0.62 [0.18–1.05]*	15.88 (3.30) *0.62 [0.15–1.09]*	14.39 (3.02) ** *0.62 [0.25–0.98]*
95%Area (mm^2^)	148.99 (102.53)	190.46 (152.39)* *0.26 [–10.1 to 10.61]*	209.30 (239.64) *0.21 [–13.14 to 13.56]*	530.12 (314.76) *0.62 [–23.99 to 25.23]*	574.78 (342.18) *0.62 [–29.91 to 31.15]*	569.81 (450.65) *0.62 [–29.24 to 30.48]*
MfAP (Hz)	0.92 (0.29)	0.96 (0.37) *0.05 [0.01–0.09]*	0.96 (0.27) *0.1 [0.09–0.12]*	1.03 (0.32) *0.31 [0.3–0.33]*	1.06 (0.27) *0.41 [0.4–0.43]*	0.98 (0.30) ** *0.23 [0.22–0.24]*
MfML (Hz)	1.49 (0.56)	1.39 (0.64) *0.25 [0.23–0.27]*	1.38 (0.63) *0.34 [0.34–0.35]*	0.96 (0.28) *0.6 [0.56–0.76]*	0.96 (0.29) *0.58 [0.53–0.61]*	0.91 (0.25) ** *0.59 [0.55–0.64]*

Each cell represents the mean (standard deviation) on top and effect size *r* [95% CI] compared to condition 1 at bottom in italics.

*Significant differences when compared to condition 1 (*p* < 0.05).

**Significant decrease when compared to condition 5 (*p* < 0.05).

*MeanAP/MeanML, Mean distance of COP in the anteroposterior/mediolateral direction; VelAP/VelML, Mean velocity of COP in the anteroposterior/mediolateral direction; *95%Area*, 95% confidence ellipse area of COP; MfAP/MfML, Mean rotational frequency of COP in the anteroposterior/mediolateral direction.*

**FIGURE 5:**
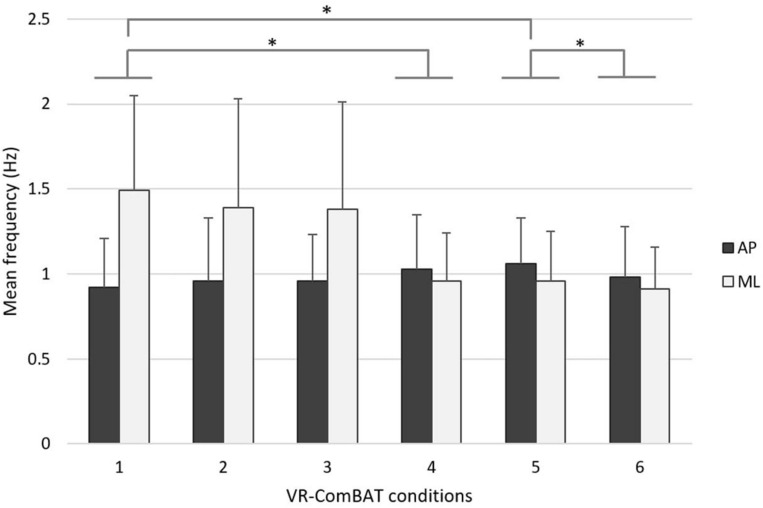
Mean rotational frequency of COP in anteroposterior (black) and mediolateral (gray) directions across the 6 VR-ComBAT conditions. *Significant differences (*p* < 0.05).

In the frequency-based analysis, an inverse trend between MfAP and MfML was observed across the conditions. Pairwise comparisons showed that MfAP increased while MfML decreased with respect to increased task difficulty (Figure 5).

## Discussion

The aim of this proof-of-concept study was to determine the safety, sense of presence, systems usability, and face validity of the newly developed VR-ComBAT in healthy young individuals. We gauged sense of presence in the VR environment and satisfaction with our novel VR-based system as important outcomes of sense of presence. We also included comprehensive quantitative comparisons of multidirectional COP outcomes across the six conditions. The results of this study support our hypotheses that the VR-ComBAT is safe, highly acceptable, and able to detect multidirectional changes of postural control in terms of COP amid different conditions from healthy young participants.

The VR-ComBAT provided a safe and feasible virtual balance testing environment. As Howarth and Costello noted (Howarth and Costello, 1997), VR environments can induce temporary side effects such as general discomfort, fatigue, headache, nausea, and irritating eyes. However, in our study, no adverse events were reported by participants. In addition, the results of SSQ suggested that the VR-ComBAT did not cause any adverse effects of VR throughout the experiment.

Our findings on the sense of presence of the VR-ComBAT measured by iPQ and SUS demonstrated that our novel VR system was well accepted by participants. The total iPQ (44.9) was 9.9-point above neutral presence (35 = 70/2), demonstrating that participants indicated being present in the VR-ComBAT environment. The means of the three subscales of iPQ all contributed to the averaged overall score [spatial presence (18.8 out of 25), involvement (11.3 out of 20), and experience realism (10.7 out of 20)]. The average total score of SUS was approximately 80 out of 100, which demonstrated high effectiveness, efficiency, and satisfaction levels of the VR-ComBAT. The high system usability of VR was also reported in a VR-based rehabilitation study (Meldrum et al., 2012).

The six conditions of the VR-ComBAT emulate an environment equivalent to the SOT. Combined, they are purported to evaluate the sensory systems involved in postural control. Comparing conditions 2, 4, and 5 versus condition 1, our findings are consistent with results from previous SOT studies in healthy younger and older adults (Cohen et al., 1996; Pletcher et al., 2017). When the somatosensory system is confronted with a challenging environment (e.g., conditions 4 and 5), healthy young participants reweighted appropriately to their vision and vestibular systems to maintain postural control. The large effect sizes observed in condition 4 vs. 1 and condition 5 vs. 1 support postural control reliance on vision and vestibular systems, respectively (Pletcher et al., 2017). When comparing condition 6 (unstable surface with VR visual conflict) to condition 5 (unstable surface with blacked out VR surroundings), the significant decreased mean velocity and mean frequency indicate that blacking out the VR surroundings compromised postural control more so than providing conflicting visual information through a moving VR surround. Our results demonstrate that the perturbed somatosensory system might be compensated for by the visual-guided scene through the VR-ComBAT. Vision contributes to balance during quiet standing, and a previous study showed that individuals with impaired vision failed to maintain their postural stability in challenging conditions (Ray et al., 2008). To this end, future studies identifying the changes of COP-based measures in patients with sensory deficits using VR-ComBAT are warranted.

In addition to the findings in the AP direction, the COP-based measures in the ML direction (e.g., MeanML, VelML, and MfML) demonstrated significant changes in this study (e.g., conditions 2, 4, 5 vs. condition 1). Specifically, the change of mean COP frequency of this study is direction-dependent where the mean frequency increases in the AP direction while it decreases in the ML direction with respect to increased task difficulty (Figure 5). While confronted with a challenging environment, healthy young adults seem to restrict ML movement to maintain postural control. This direction-dependent phenomenon was in line with current evidence that additional energy expenditure, such as muscular effort, is required to maintain lateral stability during walking (Kuo and Donelan, 2010). More evidence is required to elucidate whether there is a relationship between COP frequency changes in different directions and energy expenditure. Future studies using the VR-ComBAT will examine whether this direction-dependent phenomenon of COP frequency alters in older adults or patients with deficits in sensory integration.

A previous study that integrated VR HMD with posturography provided similar conditions as the Equitest^®^ SOT (Wittstein et al., 2020) and demonstrated a moderate reliability and a weak correlation between the developed VR SOT and the EquiTest^®^ (Wittstein et al., 2020). The VR-ComBAT of this study emulates conditions that are equivalent to those in the EquiTest^®^ as illustrated in Figure 2. In addition, our study provided a quantitative analysis of COP distance, velocity, area, and frequency in the anteroposterior and mediolateral direction. The importance of including multidirectional COP outcomes was illustrated in our direction-dependent phenomenon of frequency changes in the anteroposterior and mediolateral direction.

There are limitations to this proof-of-concept study. The current study included a relatively small number of healthy young adults. Therefore, we did not adjust for multiple comparisons in the *post hoc* analyses. Although we established the safety of the VR-ComBAT in healthy individuals, further studies are warranted to validate the VR-ComBAT in people with balance impairments. In addition, the balance testing conditions in the VR-ComBAT system were based on the testing conditions that originate from the EquiTest^®^. Although the current study demonstrated the face validity of the novel system, our new test requires validation against the EquiTest^®^. We list future directions of work to overcome the limitations observed in the current work and optimize the utilization of the VR-ComBAT. First, we plan to integrate the VR-ComBAT with a cognitive assessment to evaluate the effect of dual-tasking on reweighting for postural control. The eye-tracking integrated VR system such as HTC VIVE Pro Eye can be used to extract pupillary response, which is a valid measure of cognitive workload in demanding postural conditions (Orlosky et al., 2017). Second, we will employ the VR-ComBAT in older and/or patient populations to confirm the sensitivity of the VR-ComBAT in evaluating sensory organization of postural control. Third, not all clinical settings can afford a relatively expensive force plate system. One of the overarching goals of the VR-ComBAT development is to provide a cost-effective system. We developed the VR-ComBAT for research purposes. Thus, the cost of the current setup including force platform system ($10,000–$20,000) and VR HMD system ($500–$2,000) may be expensive for most clinics. However, we plan to create a clinically affordable VR-ComBAT by replacing the force plate with cost-effective COP measures such as Nintendo Wii Fit or gyroscope/accelerometers in smartphones. Lastly, our study found meaningful outcomes of COP changes in ML direction. Future VR-ComBAT conditions should include ML tilting in VR to further elucidate the effect of visual conflict on ML postural control.

In conclusion, this current proof-of-concept study demonstrates the safety, sense of presence, and face validity of the VR-ComBAT integrated with a COP measuring system.

## Data Availability Statement

The raw data supporting the conclusions of this article will be made available by the authors, without undue reservation.

## Ethics Statement

The studies involving human participants were reviewed and approved by IRB at University of Kansas Medical Center. The patients/participants provided their written informed consent to participate in this study.

## Author Contributions

SM and HD designed the study. SM performed the experiments. SM, C-KH, MS, and HD analyzed the collected data. All authors wrote the manuscript, discussed the results, and commented on the manuscript.

## Conflict of Interest

The authors declare that the research was conducted in the absence of any commercial or financial relationships that could be construed as a potential conflict of interest.

## Publisher’s Note

All claims expressed in this article are solely those of the authors and do not necessarily represent those of their affiliated organizations, or those of the publisher, the editors and the reviewers. Any product that may be evaluated in this article, or claim that may be made by its manufacturer, is not guaranteed or endorsed by the publisher.
